# Exploring the attitudes of healthcare professionals towards primary healthcare in northwest Syria

**DOI:** 10.1186/s12875-025-02790-5

**Published:** 2025-05-09

**Authors:** Sara Basha, Aravinda Guntupalli, Diana Rayes, Abdulkader Mohammad, Mahmoud Hariri, Lena Basha, Safwan Alchalati, Yamama Bdaiwi, Aula Abbara

**Affiliations:** 1 University of Aberdeen, Aberdeen, UK; 2 Johns Hopkins School of Public Health, Baltimore, USA; 3 Syria Public Health Network, London, UK; 4 Faculty of Medicine, University of Novi Sad, Novi Sad, Serbia; 5 Health Information System Unit, Gaziantep, Turkey; 6 Syrian Board of Medical Specialties, Gaziantep, Turkey; 7 Syria Development Centre, London, UK; 8 Imperial College, London, UK

**Keywords:** Syria, Conflict, Primary healthcare, Health system, Governance; Donors

## Abstract

**Background:**

Though primary healthcare (PHC) is an essential component of a robust health system, it remains under-developed and under-resourced in many fragile and conflict affected settings. In Syria, even pre-conflict, the health system had more emphasis on specialist and secondary care with weaker emphasis on PHC. This is beginning to change with investment from donors, international and humanitarian organisations; however, its implementation remains challenging, in part due to negative attitudes towards PHC among both physicians and patients. Our aim is to explore attitudes towards PHC in northwest Syria among relevant stakeholders.

**Methods:**

A qualitative research design using a contextualist approach was used. Semi-structured interviews were conducted with stakeholders who had experience of the Syrian health system before and after the conflict. Purposive and subsequent snowball sampling were used for recruitment. A topic guide was developed with stakeholders and interviews were conducted using Microsoft Teams. Interviews were transcribed verbatim and translated where appropriate. Inductive thematic analysis was conducted using Nvivo V.12 software.

**Results:**

Fifteen in-depth interviews were conducted; 7 were female. The main emerging themes and subthemes were: 1. Governance of the health system (subthemes: inadequate communication and coordination; the power of donors; lack of monitoring systems; inadequate health information systems). 2. The observed attitudes of community and patients’ towards primary healthcare (sub-themes: perceived patients’ attitudes towards PHC; importance of building trust with the community; impact of cost on service use). 3. Healthcare workforce and primary healthcare (sub-themes: negative attitudes towards PHC as a specialty; numbers and capabilities of healthcare professionals; changing attitudes towards PHC as a system).

**Discussion:**

Though there was some evidence that attitudes were changing, there remain prevailing negative attitudes towards PHC, including a reluctance among undergraduates to choose it as a destination specialty. Without further understanding barriers, efforts by donors and humanitarian organisations to implement effective PHC in northwest Syria may flounder.

**Supplementary Information:**

The online version contains supplementary material available at 10.1186/s12875-025-02790-5.

## Background

The 1978 declaration of Alma-Ata laid out primary healthcare (PHC) as essential to achieving healthcare for all and the 2008 World Health Report states that PHC is “the best and most affordable way to get health systems back on track” [1]. This strategy has been used successfully during health system reconstruction in post-conflict settings including Kosovo and Guatemala [2, 3]. Discussions around PHC, including in fragile or conflict affected settings are more pertinent now as we progress towards the 2030 Sustainable Development Goals of which Universal Health Coverage (UHC) is an important component and for which PHC is a key enabler [4].

In Syria, protracted armed conflict has decimated the health system through the weaponisation of healthcare, destruction of health infrastructure and the targeting and forced displacement of healthcare professionals [5]. Strains on population health and on the health system have been further affected by the protracted conflict, the COVID-19 pandemic, the February 2023 earthquakes and rising poverty rates [6]. Prior to the conflict, the health system in Syria was heavily skewed towards specialty and secondary care services which were mostly centralised to the main urban areas at the neglect of rural areas contributing to inequalities in healthcare access and inadequate utilisation of capacity [5, 6]. Though PHC existed, it was underfunded and considered to be a provider of poor-quality services to those who could not afford private healthcare [7, 8]. The heavy emphasis on secondary and specialist care in Syria has both immediate and long term implications for providing healthcare for the population [9], including increased centralisation of care exacerbating inequitable access to services with limited scope of practice that do not address broader population health issues [10]. This is also concerning given ongoing underfunding with only 25% of the humanitarian response funded in 2024 [11, 12]. As such, PHC has an essential role as a ‘gatekeeper’ for more costly secondary care or specialist services [13], and can also facilitate the delivery of a wider range of services including prevention and integration of some stigmatised but essential services such as mental health or sexual health services [14]. This is even more essential in this phase of health system reconstruction after the fall of the Syrian regime in December 2024 where streamlined, cost-effective services are needed.

In the former region of northwest Syria, which, at the time of the study encompassed Idlib and parts of Aleppo governorates, a health system had ds emerged to serve the estimated 5.1 million people in the area, of whom more than two thirds were internally displaced people (IDPs) [15]. The early withdrawal and disengagement of the Syrian Ministry of Health, during the conflict, left a void in healthcare provision necessitating local and international humanitarian organisations to step in, many of whom provide PHC [16]. In 2014 [17], the first UN Security Council Resolution on cross-border aid was passed due to interference from the Syrian government in aid destined to areas outside of its control; this allowed four border crossings to be used to circumvent government interference with aid entering directly from Turkey, Jordan and Iraq. Since then, only cross-border aid from Turkey continues with the WHO-led health cluster based in Gaziantep coordinating [10]. In an attempt to standardise and coordinate healthcare delivery in the region, the health cluster developed an Essential Health Services Package (EHSP) which aimed to cover six main intervention areas that reflected essential public health goals, providing coverage for approximately 1.6 million people through 38 facilities [18]. Four levels of PHC facilities (PHC mobile clinic, PHC unit, PHC centre and comprehensive PHC centre) have been described, each of which provides a set of preventative and curative health services appropriate for population needs. Services delivered across these four levels include child health, immunisations, nutrition, reproductive health, communicable disease, non-communicable diseases, mental health and health education [19]. An essential component of PHC delivery in the region has been community healthcare workers (CHWs) who lead health education and awareness campaigns, signpost patients to services and manage referrals within the system [20]. However, organisations often work independently with little coordination or regulation and healthcare delivery remains fragmented [10]. Bottom up initiatives within Syria developed simultaneously to address issues in healthcare governance and local actors worked to build networks and eventually independent health directorates to provide health system governance in the region; over time these committees have developed more legitimacy and become increasingly involved in coordination of international healthcare aid delivery [21]. However, establishing health system governance in the area has been challenging and remains an essential step in the early reconstruction and post-conflict rebuilding of the health system [22].

Evidence supports PHC being a backbone of a cost-effective and resilient health system [23]. However, in some contexts globally, as in Syria, negative attitudes towards PHC prevail among both relevant stakeholders and service users, possibly limiting the development and use of these services. Understanding stakeholder attitudes towards PHC is essential when planning development of the PHC system to understand contextual needs and ensure stakeholder buy-in. Our aim is to explore the attitudes and perceptions of healthcare professionals and other relevant stakeholders towards PHC in northwest Syria.

## Methods

A qualitative study design taking a contextualist approach [24] was chosen, using semi-structured interviews with key stakeholders with experience of PHC in northwest Syria during the armed conflict. Individual semi-structured interviews were most suited to explore in detail the unique ideas and perceptions of interviewees with an invested interest in the development of healthcare in this fragile context [24]. They also provided an opportunity for more in-depth interviews and for the key informants to be able to speak more fully to their experiences. We define PHC as per the WHO definition "PHC is a whole-of-society approach to health that aims at ensuring the highest possible level of health and well-being and their equitable distribution by focusing on people’s needs and as early as possible along the continuum from health promotion and disease prevention to treatment, rehabilitation and palliative care, and as close as feasible to people’s everyday environment" [25].

### Participants

Participants were selected based on the inclusion and exclusion criteria shown in Table 1. A two-stage sampling process was used; purposive sampling to select participants with rich insight into the topic, followed by snowball sampling to increase the range of participants.

**Table 1:** Participant inclusion and exclusion criteria used for recruitment

Inclusion criteria	• Age 18 years and over and able to consent. • Health professionals and other stakeholders including hospital, PHC or program managers; humanitarian medical professionals; those working with international organisations; those involved in the planning of services and funders who have experience using, planning or delivering PHC in northwest Syria after the onset of armed conflict.
Exclusion criteria	• Unable to interview virtually

Participants were sent the participant information sheet and consent form by email; all queries were answered prior to interview, and written or verbal, recorded, consent taken. Participants were aware that participation was voluntary, and that information provided would be encrypted, stored securely and anonymised completely. Permission for audio recording was sought and granted. Interviews were recorded using the recording function on Microsoft Teams with audio only to limit participant identification.

### Data collection

Interviews were conducted, using Microsoft Teams, in English or Arabic (as per participant preference), by SB, independently or supervised by AA. SB is a female doctor trained in qualitative research; she works in the United Kingdom (UK) health system where primary care (or general practice as it is called in the UK) is well established and practitioners are respected. Interviews continued until theoretical saturation was reached, the point at which additional data failed to generate new information [24]. Transcribing and preliminary analysis was carried out alongside data collection to ensure that new interviews were generating additional insights. Once emerging themes were found to be repetitive, data collection was concluded.

Each interview lasted 22 to 120 minutes with a mean of 67 minutes. Interview length varied primarily due to time restraints of interviewees, any participants that felt they had more information to add following the interview were able to contact the research team to further discuss. A topic guide, found in appendix A, was developed based on the literature and stakeholder consultation to ensure questions were applicable whilst allowing for exploration of new concepts. Initial broad literature review revealed very little research relating to PHC in conflict settings, however highlighted some important concepts to include in questioning such as cost effectiveness and task shifting. Questions were developed based on this review as well as our research question then reviewed with stakeholders in the Syrian health system with experience working in managerial positions within NGOs in Syria.

Audio recordings were uploaded to Nvivo V.12 and transcribed verbatim, translated from Arabic to English where needed, then analysed. Interviews were continued until no new themes emerged.

### Analysis

Inductive thematic analysis, to allow for the development of themes from the bottom up to minimise the impact of the research teams personal attitudes towards PHC on analysis [24], was conducted following the six steps recommended by Braun and Clarke [26] using Nvivo V.12 software. Open coding was used to systematically categorise each transcript and themes were developed from these codes. Three procedures [27] were used to minimise bias throughout analysis: constant comparison, negative instances and rival thinking. Initially using open coding to break down data before reassembling it into higher level codes and themes necessitated constant review of different ideas and patterns and interrogation of presumptions of the data. Utilising the support of expert team members to review this process allowed for groupings and coding to be challenged and adjusted accordingly. Themes were discussed with the research team as a form of triangulation to strengthen analysis [24]. Reporting adheres to SPQR (Standards for Reporting Qualitative Research) [28].

## Results

### Participant characteristics

15 participants (8 men) were interviewed; 9 were recruited through purposive sampling and 6 through snowball sampling. All were Syrian, though more than half were located outside of Syria at the time of the interview. Most were undergraduate or postgraduate doctors though of the latter, most worked in humanitarian organisations or in managerial positions in healthcare. Participant characteristics can be found in table 2.

**Table 2:** Participant characteristics including sex, age, cadre and location

**Participant ID**	**Sex**	**Age**	**Cadre**	**Current Location**
1	Male	51-60	Health service manager, trained doctor	Turkey
2	Male	31-40	Field medical coordinator, trained doctor	Syria
3	Female	41-50	Humanitarian organisation; trained doctor	Turkey
4	Male	31-40	Global health researcher, trained doctor	USA
5	Male	31-40	Humanitarian organisation; trained doctor	Syria
6	Male	21-30	Medical Student	Syria
7	Male	21-30	Medical Student	Syria
8	Female	21-30	Doctor	UK
9	Male	21-30	Doctor	UK
10	Female	31-40	Global health researcher, trained pharmacist	UK
11	Male	31-40	Medical education coordinator, trained doctor	Syria
12	Female	31-40	Doctor	Syria
13	Female	41-50	Doctor	UK
14	Female	31-40	Pharmacist	UK
15	Female	31-40	Doctor	UK

### Key themes

**Fig. 1:**
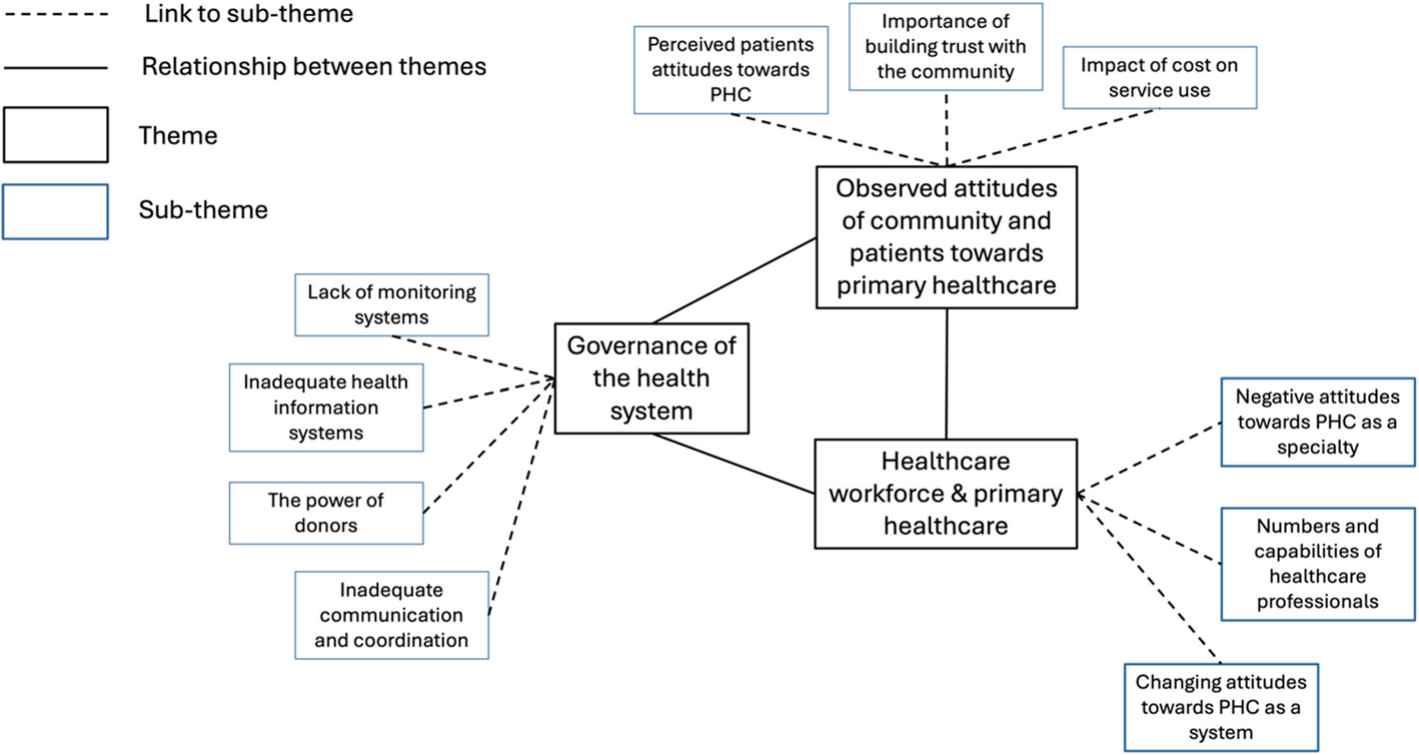
This figure shows the three key themes and their related sub-themes identified from the thematic analysis of the interviews

Thematic analysis identified three overarching themes: governance of the health system, observed attitudes of community and patients towards PHC and healthcare workforce and PHC (Figure 1).

#### 1. Governance of the health system

Concerns around health system governance in northwest Syria was a strong theme with participants discussing its impact on PHC functionality; subthemes are discussed below.

##### 1a. Inadequate communication and coordination

Participants described the impact of inadequate communication and coordination amongst and between humanitarian organisations and local health directorates in northwest Syria. Organisations were seen to work in silos leading to mismanagement of resources and discrepancies in services. Participants also expressed concern regarding the absence of standardised healthcare delivery, particularly in terms of disease management protocols and standards for PHC. Increasing local health directorates’ authority was suggested as a way to streamline service delivery in PHC, avoid duplication and standardise services. A participant noted:

“…there isn’t any coordination between different organisations, so you can find in one village there are 2 primary healthcare facilities and in 20-30 villages you won’t find any. Our problem is that they are not communicating,” – (P9, male, Doctor)

Inadequate communication extends to local actors, with several participants explaining that organisational aims and objectives were not communicated effectively to healthcare professionals in the field. Subsequently, there is misunderstanding and lack of confidence in projects being implemented and therefore a lack of buy-in and commitment to effective service delivery amongst health care professionals as the following participant noted:

“our problem is that they are not communicating, they are not telling us what the aim is, what the benefit of the project is or what our future plan is. (…) Subsequently, when doctors are implementing these projects or working in it they are doing it reluctantly”. – (P9, male, doctor)

##### 1b. The power of donors

Many participants described how donors had ultimate control over healthcare service development and delivery, often without appropriate consideration of community needs, resulting in a ‘top-down’ approach to service delivery. Concerns were raised around time limited funding, minimising effectiveness of PHC and impacting job security; this has contributed to high staff turnover and poor morale. These factors foster a sense of lack of control amongst doctors, possibly influencing service delivery and perceptions of PHC. A participant noted:

“They want interventions that are fast, that can be completed in the fastest time with the least costs and they don’t care about the type….. The healthcare workforce always feels anxious, always threatened to lose their source of income if the funding is not renewed.” (P8, female, doctor)

However, this power is, to an extent, holding healthcare professionals accountable through a functional complaints system. One participant explained the change that this has brought about in the empowerment of patients, allowing them to demand their rights from health service providers:

“if a staff or a person running an organisation does any mistake or, I don’t know, any mistreatment, immediately the social media is full of that. Feedback mechanisms are there and people know how to use them.” – (P3, female, NGO worker)

##### 1c. Lack of monitoring systems

The importance of functional quality assurance systems was highlighted by a number of participants, particularly those in managerial positions; however, the difficulty of achieving this without a unified governing body was stressed. Inadequate monitoring systems have resulted in the employment of uncertified doctors and medical students as doctors, and extreme variation in practitioner capabilities. Corruption in the health service was discussed, a further ramification of ineffective quality assurance systems. Participants noted:

“There were a lot of scam cases, anyone can just print a diploma and tell you I’m a doctor and he doesn’t even know how to write his name in English.” – (P3, female, humanitarian organisation employee)

“Who’s doing the quality control, who is doing this monitoring role and who is playing this liability responsibility role that really can have an impact on the quality and follow up. (...) What about medical errors, who follows this up?” (P4, male, global health researcher)

##### 1d. Inadequate Health Information Systems

The inability to collect, store, manage and process data was identified as a significant limiting factor in PHC by a number of participants. Challenges surrounding following up patients, referring and taking a holistic approach to patient care were discussed across numerous interviews. There are no electrical records used in PHC and no ability to send comprehensive patient information when referring patients. This adversely affects service delivery and, in some cases, puts patients at risk by relying on them to transfer their own case from one doctor to the next.

*“the patient has to be aware and have his documents, keep the last ECG (electrocardiogram) with him, keep the last medications he has been on and memorise his medications. If he is not very well educated, he will go to the doctor and tell his doctor ‘doctor I am taking the drug that is in the box with a yellow and red lid that’s shape is a tube and it has a bird on its side from the company x’ for example” – (P1, male, health service manager)*

#### 2. The observed attitudes of community and patients’ towards primary healthcare

Challenges and opportunities around patients’ perceived attitudes towards PHC were discussed in all interviews with three sub-themes identified: percieved patients’ attitudes towards PHC, importance of building trust with the community and impact of cost on service use as expressed by the participants.

##### 2a. Perceived patients’ attitudes towards PHC

In northwest Syria, utilisation of PHC increased after the onset of the conflict. New facilities and lower delivery costs compared to secondary or specialist care improved accessibility and utilisation of PHC in the region. However, participants agreed that not all patients are convinced, with many still preferring secondary or specialist care if offered the choice. PHC pre-conflict as run by the Syrian Ministry of Health was almost unanimously agreed to be of very poor quality with limited services leading to distrust of the system and limited understanding of its role. Emphasis was placed on the importance of public education, building trust and promoting PHC to improve perceptions. Participants noted:

“we can say that it’s half half. 50% have developed this understanding and the other 50% have not and will go straight to specialists.” – (P2, male, field medical coordinator)

“the main reason [patients lack faith] is that they think it is bad quality, it's very low quality.” – (P10, female, global health researcher)

##### 2b. Importance of building trust with the community

Many concerns regarding patients’ perceived inappropriate use of PHC services were linked to likely distrust amongst the community, as mentioned previously. Participants explained that many services provided through PHC, such as mental health support, child protection and community health professionals, did not align with the communities’ beliefs and preferences and so were ineffective due to limited acceptance. The importance of building trust with the community and increasing health promotion and community engagement to mitigate these issues was emphasised.

“If I come to provide a service that is not appropriate, I won’t benefit from it. There is significant funding going into things that are not achieving the results that we want. Things like child protection and mental health honestly, I think people are not ready for these ideas. People first need the basics, they need you to ensure the basics, then people may be prepared to accept these things.” – (P2, male, field medical coordinator)

##### 2c. Impact of cost on service use

A recurring subtheme was the effect of free services on patients’ attitudes towards PHC. Although several participants acknowledged the importance and benefit of providing free services, many felt it may also be contributing to abuses of the PHC system. Free services are generally perceived to be low in quality and value, leading to patients using services flippantly. This overwhelms PHC, limiting its benefit and contributes to the misuse of secondary care. The absence of payments, as well as available feedback mechanisms, may have created an entitlement amongst patients. Patient demands can be inappropriate, including excessive antibiotic demands, and resistance by healthcare professionals can be met with verbal and physical abuse. Participants noted:

“because it is free, they like to get medication, come to the dispensary and be seen, measure their blood pressure, get themselves checked up.” – (P5, male, humanitarian organisation employee)

“they would come to us shout at us that you should give me antibiotic, I demand you to give me antibiotics” – (P3, female, humanitarian organisation employee)

#### 3. Healthcare workforce and primary healthcare

Interactions of the healthcare workforce and PHC were discussed in all interviews with three sub-themes identified: negative attitudes towards PHC as a speciality, numbers and capabilities of healthcare professionals and changing attitudes towards PHC as a system.

##### 3a. Negative attitudes towards PHC as a speciality

All participants agreed that there is a longstanding negative perception of family physicians in Syria. Prior to the conflict, general doctors in Syria were doctors with a couple years of postgraduate training and no specialism; family medicine did exist as a speciality but was limited with very few doctors choosing this training pathway. Participants noted:

“Primary healthcare to us was taught as the speciality that had the lowest grades, the person that didn’t get any other speciality in matching would go to primary healthcare.” – (P9, male, doctor)

Support around developing PHC speciality training was not unanimous. Several participants, particularly practitioners working in the field, suggested priority should be placed on training more specialists instead. Of particular concern were the attitudes of medical students interviewed who lacked an understanding of the PHC specialist’s role; one believed a PHC speciality is unnecessary and the other suggested that specialising in PHC should be a stepping stone to other specialities. Participants noted:

“Frankly, if specialist doctors are available in general, specifically internal doctors, children, women, there is no need for doctors to specialise in primary health care. For example, a paediatrician can work in primary care centres and manage children, they can also work in a specialist clinic and at the same time in a hospital depending on what is available to them. As a doctor, it is better to specialise and work in a hospital than it is to specialise in general specialisation and work in a primary care centre.” – (P7, male, medical student)

Participants in favour of developing this speciality stressed the importance of making it more desirable with financial incentives, improving prestige, improved working hours and by increasing PHC placements and teaching in medical schools. Participants noted:

“Firstly, there needs to be incentives for PHC so that people can specialise in this. So, someone needs to have the same salary as other doctors. Secondly, they need to have extra benefits like a weekend off for example.” – (P11, male, medical education coordinator)

##### 3b. Numbers and capabilities of healthcare professionals

Participants noted that working in PHC is incredibly challenging due to the overwhelming case load. Overstretched services minimise time spent in consultations, limiting physicians’ abilities to appropriately assess and manage patients. Time pressures and patient demands cause some doctors to inappropriately prescribe medications to avoid confrontation and shorten appointments. These challenges are exacerbated by poor quality medical education and limited professional development for clinicians. Numerous participants stressed the importance of providing long term capacity building with appropriate follow up monitoring for physicians in PHC. Participants noted:

“when you see 200 patients a day, 300 patients a day, if I have like in 24 hours I am seeing 800 patients, this is inhumane.” – (P4, male, global health researcher)

“no one taught me how to work, what are the things that I should transfer to hospital, what are the things I should manage myself, what are the things I should transfer urgently.” – (P9, male, doctor)

##### 3c. Changing attitudes towards PHC as a system

Participants explained that pre-conflict understanding of PHC was very limited due to the minimal teaching on the subject and the dysfunctional system. The attitudes of some healthcare professionals in northwest Syria towards PHC have changed with the development of some functional PHC services, primarily due to their triage role for secondary care. However, some participants felt that many doctors were not yet convinced, only participating out of necessity. Developing commitment to PHC in Syria is challenging and was not always perceived as a priority. Participants noted:

“I think right now, specialised healthcare professionals recognise the importance of primary healthcare otherwise, they would be overwhelmed.” – (P4, male, global health researcher)

“Until now, a lot of my friends still say, why are the organisations funding primary healthcare facilities so much, what’s the point in them? They should be funding the hospitals more, we are in a greater need” – (P9, male, doctor)

## Discussion

To our knowledge, this is the first study which examines attitudes towards PHC in northwest Syria prior to the fall of the Syrian regime in December 2024 from the perspective of service providers. Our study highlights some of the perceptions around PHC which are important to understand and address where possible, particularly given the importance of PHC as Syria's health system rebuilds. Without addressing these, negative attitudes may continue, hampering efforts to strengthen PHC in this crucial phase. Strengthening PHC is essential across all geographical areas of Syria given increasingly scarce resources, high population health needs and the need to provide effective, equitable care both now and in the early reconstruction phase [14]. A strong PHC system can form the basis for a functioning health system and support essential services including health promotion and prevention which are relevant to population health e.g. screening, vaccination during and after conflict [29, 30]. However, both in Syria and other contexts, negative attitudes towards PHC persist [31, 32] though, with concerted efforts and promotion from the World Health Organisation (WHO) and other stakeholders to emphasise the importance of PHC among both service providers and service users, this is can be changed [33].

Other than investment and strengthening governance around the planning and introduction of services, promotion of PHC among the community, medical students and current doctors is an essential interim measure. Medical students interviewed expressed reluctance to consider training in PHC as a speciality. Several factors can contribute to this reluctance including a limited understanding of the breadth of a PHC physician’s role, absence of PHC physician role models and few or no meaningful experiences in primary care [34]. Limited exposure to and subsequent limited understanding of PHC in the undergraduate curriculum can be addressed by introducing either short intensive courses or longer term programmes and placements focused on primary care as has been done in a variety of settings including the United States, Australia, News Zealand, Pakistan, Hong Kong and numerous European countries [35]. These programmes have been found to improve understanding of primary health care and increase uptake of primary care physician roles [36, 37]. Another possible contributing factor could be the lack of established pathways for PHC training such as exists in the United Kingdom for General Practitioners where a three year program of rotations through relevant specialities e.g. emergency medicine, paediatrics, psychiatry is provided [38] or the four year Family Medicine residency programme available in Saudi Arabia allowing candidates to rotate between 13 specialities [39] Some in Syria might confuse a PHC physician with a ‘generalist’, who is usually someone who has done 1-2 years of non-specialist training after medical school and, either through choice or lack of opportunity, not entered specialist training [40], contributing to the lack of faith in these doctors. Discussions are currently under way as to how to establish or reinforce training programs for PHC physicians in t Syria by various academic, state and non-state actors with support from the newly established Ministry of Health.

Strengthening training in PHC and attracting the best candidates is also essential to improve the profile of PHC. Despite promising innovations and efforts among various actors including humanitarian organisations and SBOMS in northwest Syria, it has been challenging to provide quality, specialist training for a variety of reasons. Though this is gradually changing, there has traditionally been an onus placed on individuals to be predominantly responsible for their own training [41]. In many contexts globally, training for work in PHC, staffed mainly by non-PHC specialists, is inadequate [42] limiting the system’s capabilities [42]; this can contribute to challenges in developing and recruiting PHC physicians [43]. Possible solutions suggested by participants included increasing teaching and exposure to PHC during undergraduate education, a valid method of increasing uptake of the speciality [35, 44], capacity building for PHC staff, promoting the speciality among both service users and providers and providing financial incentives for PHC physicians. Changing perceptions of PHC among healthcare professionals has been successful in other contexts such as the United Kingdom, United States [37] and Lithuania [45, 46].

Promoting PHC among the community is also an important means of improving engagement and trust. Participants described the perceived lack of faith in the pre-existing PHC system and limited understanding of the role of PHC as potential reasons for patients’ dismissal of the system. Prior to the conflict, PHC consisted mainly of immunisation clinics and some maternal and child health services [47]. This along with the general concept that ‘generalist’ doctors were those who could not specialise due to a perceived lack of competence resulted in a poor perception of PHC by the community and a poor understanding of the role of a primary care physician. Though increased availability of PHC services during the conflict in northwest Syria has increased utilisation (perhaps due to the lack of another choice) more work is needed to change community perceptions. This could be through community engagement initiatives to ensure services are responsive and appropriate to local needs and through community advocates and education [48]. In addition, in northwest Syria, most healthcare provided by humanitarian actors is free, something which is appropriate as more than 90% of the population live in poverty [49]. However, as seen in other contexts, when free healthcare is provided, it may affect the perceived worth patients place on it [50]. Participants felt that cultural attitudes towards free services may be leading to an abuse of PHC and fostering a sense of entitlement amongst patients; this could for example contribute to antibiotic misuse, a recognised issue in Syria [51]. Introducing service charges was suggested by some participants and has been found to decrease healthcare over utilisation in other contexts [52]. However, in this context, it may also carry the risks of creating further barriers to essential healthcare services [52]. Further enquiry is necessary to understand the nuances of this issue and determine the most appropriate approach.

Tackling poor perceptions of PHC in Syria also requires the strengthening of healthcare governance, a concern raised by several participants. These issues affect most parts of the health system in northwest Syria where the poor coordination and the absence of long-term planning, negatively affect equitable distribution of services, appropriate to local community needs [22]. Poor coordination and communication among humanitarian actors is not unique to this context and is noted to contribute to ineffective use of resources, duplication and incomplete coverage [53]. This can also negatively affect participation of local actors and the community in health system development [54]. Furthermore, the absence of effective accountability mechanisms was discussed by numerous participants, an issue identified in previous studies [22]. The forced exodus of healthcare professionals, many of whom were senior, could also adversely affect training and supervision, contributing poor quality services and the inability to assess service delivery [55]. Notably, participants indicated that social accountability mechanisms are functional in northwest Syria; this recognised method of highlighting local problems and structural issues and could be capitalised on to improve PHC delivery [56].

Further work which explores how attitudes and structural challenges related to PHC and how they can be addressed is needed, particularly in this new phase in Syria's health system. From this work, it is clear that promoting PHC as a specialty among students, health professionals and patients is essential; this can be through working with organisations or entities which have successfully implemented such changes in similar contexts. Investment in, expansion of and standardisation among existing PHC staff and facilities is also key as part of a systems strengthening approach; this can include standard operating procedures, training and adequate resourcing to support an expansion of services which go beyond the delivery of primary care. This can allow PHC centres to support community and health promotion initiatives, vaccination and screening; these would reduce pressure on other parts of the health system.

### Limitations

This study focused on the perceptions of health professionals and other stakeholders; however, future work which focuses on that of patients and the wider community is essential to better understand the issues and how they can be addressed. We also note an imbalance in cadre (mainly doctors) and future work should engage more with other healthcare providers. Recruiting these was challenging due to fewer links and less readiness to participate as seen in other studies. This study focuses on attitudes in northwest Syria and though there may be similarities in other areas of Syria and other similar contexts, some contextual issues may be less generalisable. Future work should work to overcome these limitations and to include more focus on CHWs and other cadres which are core to PHC delivery.

## Conclusion

This study highlights some of the challenges to establishing and maintaining an effective PHC system as reported from the perspective of stakeholders. Negative perceptions, particularly among medical students, are concerning for their potential future reluctance to enter PHC training even if established programs could be provided. As such, addressing issues around governance, quality of care and enhancing opportunities for more formalised PHC training while promoting PHC among both health professionals and the community are essential steps to support effective PHC programming.

## Supplementary Information

Supplementary Material 1.

## Data Availability

It is not possible to share data collected during the interviews as recordings have been destroyed as per the ethics agreement. Transcriptions cannot be shared as this data was collected during active conflict; as such there are sensitivities around its sharing. Upon reasonable request a redacted version of coded transcripts may be shared.

## Abbreviations

PHC: Primary Healthcare
UHC: Universal Health Coverage
IDPs: Internally Displaced People
UK: United Kingdom
SPQR: Standards for Reporting Qualitative Research
USA: United States of America
ECG: Electrocardiogram
WHO: World Health Organisation
SBOMS: Syrian Board of Medical Specialists
