# Supplementary material for: Exploring the attitudes of healthcare professionals towards primary healthcare in northwest Syria
Source: BMC Prim Care. 2025 May 9;26:151. doi: 10.1186/s12875-025-02790-5 (PMC12063337; doi:10.1186/s12875-025-02790-5)
Supplement: Supplementary file 1 — Supplementary Material 1. [file 12875_2025_2790_MOESM1_ESM.docx]

**Appendix A:**

**Semi-Structured Interview Guide – KEY INFORMANT INTERVIEWS**

| ***STUDY TITLE: Perceptions and attitudes of primary health care in Syria among healthcare workers and other relevant stakeholders*** | | | |
| --- | --- | --- | --- |
| *Interview Type* | ***Key Informant****/Individual* | | |
| *Participant(s) ID* | *_________________* | | |
| *Interviewer ID* | *_________________* | | |
| *Interview Date* | *___ (DD) / ___ (MM) / _______ (YYYY)* | | |
| *Interview Time* | *______ (Hour) / ______ (Minute) AM/PM* | | |
| *Interview Duration* | *______ (Minutes)* | | |
| *Interview Language* | *___Arabic ___English ___Other______________* | | |
| *Data Recording* | *___Audio-recorded* | | |
| ***PARTICIPANT INFORMATION*** | | | |
| *Participant Age* |  | | |
| *Participant Sex (Observed)* | *__Female ___Male* | | |
| *Participant Role* |  | | |
| *Participant Location* |  | | |
| ***COMPLETE AFTER INTERVIEW*** | | | |
| *Participant Questions and Feedback* |  | | |
| *Interviewer Observations and Feedback* |  | | |
| *Interviewer Questions about Interview* |  | | |
| *Problems or Adverse Events* | *Description of Problem:* | *Steps Taken:* | *Resolution:* |
| *Field Supervisor Review (Check when complete)* | *___ Audio-transcription*  *___ Informed consent form signed by interviewer* | | |

*Thank you for agreeing to participate in this study. The aim of our study is to understand the attitudes and perceptions of primary healthcare in Syria among health professionals and other stakeholders who have experience of working in Syria’s health system after the onset of conflict. This is to explore the potential barriers surrounding the development of a primary healthcare system and how they may be overcome as well as current areas of good practice to facilitate the shift to primary healthcare.*

**PART 1. INTRODUCTION**

*I will start by asking you a few questions about yourself and your work.*

1. Can you tell me a little bit about yourself?

*Probes:*

- - *What is your role?*
  - *What are your main responsibilities?*
  - *Do you work with an international or local organisation?*
  - *How have you interacted with the Syrian health system?*

1. Can you tell me about your previous work experience?

*Probes:*

- - *What kinds of services have you provided?*
  - *Which populations do you work with?*
  - *Where has your work primarily been (geographical area, public/private sector)?*

**PART 2. PERCEPTIONS OF PRIMARY HEALTHCARE**

*I will now ask you what your thoughts are regarding primary healthcare in Syria, what has it been like in the past, how has it been used throughout the conflict and what you think the benefits and issues are around it.*

1. What is primary healthcare?

*Probes:*

- What do you understand by the term primary healthcare?
- What are the advantages of having a primary healthcare system in place?
- What are the disadvantages of having a primary healthcare system in place?

***PRIMARY CARE SERVICES BEFORE THE CONFLICT***

1. What was primary care like in Syria before the onset of the conflict?

*Probes:*

- *Service delivery: Tell me about the types of services that were available and the coverage that these provided.*
- *Service quality: What do you think about the quality of primary care services in Syria before the conflict?*
- *Service utilisation: Do you think that patients made use of primary care services before the conflict? (if not, why?)*

***PRIMARY CARE SERVICES DURING THE CONFLICT***

1. Can you tell me about the primary care services that have been available in Syria during the conflict?

*Probes:*

- *Service delivery: Tell me about the types of services that are available and the coverage that these provided.*
- *Service cost: are all primary care services public, are there any informal payments involved and does this affect perceptions or utilization of primary care by service users?*
- *Service quality: What do you think about the quality of primary care services in Syria throughout the conflict?*
- *Service utilisation: Do you think that patients have made use of primary care services during the conflict? (if not, why?)*
  - *Do you know any specific organisations that have been able to provide effective primary care?*
  - *Have there been any improvements in primary care services after the onset of the conflict compared to before?*

1. How is primary care integrated into the overall health system in Syria?

*Probes:*

- *What are the referral pathways between primary and secondary care like?*
- *Can you tell me about communication between primary and secondary care practitioners?*
- *Are primary healthcare services conveyed as first line to service users?*

1. Do you think that secondary care services reduce the need for primary care services in Syria?

*Probes:*

- *Do you think that the relationship between primary and secondary care has changed since the onset of the conflict?*
- *Do you think that there is a preference towards secondary care amongst service users?*
- *Is primary healthcare utilised by certain demographics more than others? (e.g rural populations or people who cannot afford private healthcare)*
- *How is funding and foreign aid prioritised when allocated to the health system?*

***PRIMARY HEALTHCARE PROFESSIONALS***

1. What is your impression of the skills and abilities of primary healthcare professionals?

*Probes:*

- *Can you tell me about the training of primary care physicians?*
- *What types of services do you think primary care physicians should be able to provide?*
- *What is your understanding of the other healthcare roles necessary in primary care?*

1. Do you think that there is a need for specialist doctors trained in general practice/family medicine?

*Probes:*

- *Is there or has there ever been a specialty training programme for general practice/family medicine in Syria before?*
- *What aspects of primary care may discourage graduate doctors from pursuing a career in general practice/family medicine?*

**PART 3: WHAT CAN BE DONE TO IMPROVE THE CURRENT SITAUTION**

*Now, I will ask you about the healthcare system moving forward and your ideas around incorporating primary care services into this.*

***THE NEED FOR PRIMARY HEALTHCARE***

1. Do you think that primary healthcare should be a priority when rebuilding the Syrian healthcare system?

*Probes:*

- - *What is the relevance of primary care services in Syria? (e.g universal healthcare coverage)*
  - *Do you think that developing primary healthcare will be cost-effective in Syria?*
  - *Is there any other value to PHC than its cost-effective nature?*

***AREAS FOR CHANGE***

1. What changes do you think, if any, should be made to the current primary healthcare system in Syria?

*Probes:*

- - *Do you think training for primary care physicians could be improved?*
  - *What services could benefit from integration into primary care in Syria?*
  - *Are there any areas within the relationship between primary and secondary care in Syria that need to be changed?*
  - *What is your opinion on task shifting in primary care?*

**CLOSING**

1. Is there anything else you would like to say, or you feel we should know to understand the primary healthcare service in Syria?
2. Do you have any questions for us?
3. Are there any other individuals you think we should speak with to learn about their perspectives?
4. Would you be willing to be contacted for a follow-up interview?

***Thank you very much for your time.***

***Definitions:***

- *Primary healthcare:*
  - *"PHC is a whole-of-society approach to health that aims at ensuring the highest possible level of health and well-being and their equitable distribution by focusing on people’s needs and as early as possible along the continuum from health promotion and disease prevention to treatment, rehabilitation and palliative care, and as close as feasible to people’s everyday environment." WHO and UNICEF.*
  - *Healthcare provided in the community for people making an initial approach to a medical practitioner or clinic for advice or treatment. Oxford Language.*
- *Secondary healthcare:*
  - *Secondary Health Care is the specialist treatment and support provided by doctors and other health professionals for patients who have been referred to them for specific expert care, most often provided in hospitals. International Medical Corps UK.*
- *Tertiary healthcare:*
  - *Care for people needing complex treatments. People may be referred for tertiary care (for example, a specialist stroke unit) from either primary care or secondary care. NICE.*
  - *Highly specialized medical care usually over an extended period of time that involves advanced and complex procedures and treatments performed by medical specialists in state-of-the-art facilities. Merriam-Webster.*
- *Universal healthcare coverage:*
  - *UHC means that all individuals and communities receive the health services they need without suffering financial hardship. It includes the full spectrum of essential, quality health services, from health promotion to prevention, treatment, rehabilitation, and palliative care across the life course. WHO.*

**Appendix B (translated to Arabic):**

**CONSENT FORM**

**Perceptions and attitudes of primary health care in Syria among healthcare workers and other relevant stakeholders**

Please **initial** each box:

1. I have read and understand the participant information sheet version 2 and date 24/06/21 for the above study.
2. I have had the opportunity to consider the information, ask questions and have had these answered.
3. I understand that my consent is voluntary and that I am free to withdraw my consent at any time up to the start of analysis without giving any reason.
4. I consent to providing my contact details to the research team where it is relevant to this research.
5. I understand that non-identifiable data will be stored on secure computers in the University of Aberdeen and identifiable data will be stored securely as hard copy on University premises. I understand that if I choose to withdraw, data collected prior to this may still be used.
6. I understand that data collected during the study may be looked at by individuals from the University of Aberdeen, it is relevant to this research. I give permission for such access.
7. I agree to be contacted for clarification or other issues related to the interview.
8. I understand that audio recording will be undertaken and give permission for this.
9. Agree to have anonymised quotes from the interview used in study reports, publications or presentations
10. **I consent to taking part in the above study as an interviewee.**

**Name of PARTICIPANT** ………………………………………………………………………………………………………

Signature………………………………………………………………………. Date …………………

OR

Verbal consent recorded:

**Name of PERSON TAKING CONSENT**…………………………………………………

Signature………………………………………………………………………. Date …………………

**Appendix C (translated to Arabic):**

# Information for research study participants

***Perceptions and attitudes of primary health care in Syria among healthcare workers and other relevant stakeholders***

We would like to invite you to take part in a research study. Before you decide, you need to understand why the research is being done and what it would involve. Please take time to read the following information carefully. Talk to others about the study if you wish. Ask us if there is anything that is not clear or if you would like more information. Please take time to decide whether or not you wish to take part.

**Introduction**

Primary healthcare is essential for more equitable and comprehensive delivery of healthcare across populations and is vital in low resource settings. When looking at rebuilding the health system(s) in Syria, which have not only been devastated by conflict but also the COVID-19 pandemic, strong consideration must be given to primary healthcare as their backbone. As such, as we look to the early reconstruction phase of Syria’s health system, understanding attitudes towards primary healthcare in Syria are important as a means of understanding potential barriers and how they may be overcome. This is essential as even if a primary health care system and infrastructure were developed and improved, there may be other barriers for services users to access services.

**What is the purpose of this study?**

This research aims to understand the attitudes and perceptions of primary healthcare in Syria among health professionals and other stakeholders who have experience of working within Syria’s health system, for example those working directly in healthcare roles, advisory positions or funders etc., after the onset of conflict. This is to explore the potential barriers surrounding the development of a primary healthcare system and how they may be overcome as well as current areas of good practice to facilitate the shift to primary healthcare

**Why have I been chosen to take part?**

You have been asked to take part in this project as you have been identified as a relevant stakeholder in the Syrian healthcare system due to your work within that system either as a healthcare worker, humanitarian worker, or a person involved in the planning of services or funder with experience working on Syria’s health system after the onset of the conflict.

**Do I have to take part?**

No, you do not have to take part. If you initially think you would like to participate but change your mind at any time, tell us and we will withdraw you from the study immediately.

**What does taking part involve?**

If you agree to participate in this study, one of the researchers involved will contact you to perform a 60-minute interview on Teams to ask about your ideas regarding primary healthcare. The interview will include questions and prompts about your opinion regarding the provision of primary care, your impression of primary care practitioners and your ideas surrounding the acceptability and accessibility of primary healthcare for patients in Syria. The aim is to produce a report which will highlight the ideas that participants have discussed. All information will be anonymised and not traceable to any of the participants.

We will ask you for your consent to participate in the interview before we begin. We will request to audio record the interview in order to transcribe it later and store it in an anonymised way in a password protected file on the University of Aberdeen OneDrive that will only be accessible to the research team. The interview will be transcribed and translated into English (if applicable) by the student, Sara Basha. The interview recording will be destroyed after the analysis has been completed, however, the anonymised transcription will be stored in the University of Aberdeen secured, password protected One Drive for up to 10 years.

**What are the risks associated with my taking part in the study?**

There is no risk in taking part in this study. The interviews will be held online through the Teams platform with is encrypted and all collected data will be anonymised so there is no risk of identification of participants.

**What are the benefits of taking part in the study?**

By taking part in this study, you will be helping us understand better the current attitudes and perceptions around primary healthcare in Syria which will be useful in the transition towards the development of a primary healthcare service in Syria.

**Will my taking part in this study be kept confidential?**

If you consent to take part your data related to this study may be inspected by the other researchers involved in this project, Dr Aula Abbara and Dr Aravinda Guntapali to check that the study is being carried out correctly. All information which is collected during the course of the research will be kept strictly confidential and will have all identifying information removed.

**What will happen to the data you get from me?**

The University of Aberdeen will act as the data controller for this study and will be responsible for looking after data and using it properly. Your rights to access, change or move this information are limited, as we need to manage the information in specific ways in order for the research to be reliable and accurate. To safeguard rights, we will use the minimum personally-identifiable information possible. For more information visit <http://www.abdn.ac.uk/privacy> or contact the University of Aberdeen Data Protection Officer.

The University of Aberdeen will collect information from you for this research study in accordance with our instructions. We will use your name and the contact details you provide to contact you about the research study, and make sure that relevant information about the study is recorded and to oversee the quality of the study. The only people in the University of Aberdeen who will have access to information that identifies you will be people who need to contact you to arrange study visits or audit the data collection process. The people who analyse the information will not be able to identify you and will not be able to find out your name, address or contact details*.* The University of Aberdeen will keep non-identifiable data about you on its computers and identifiable information about you as hard copy, for 10 years after the study has finished.

**Who is organising and funding the study?**

The study is being organised by the University of Aberdeen. There is no funding for this study.

**What will happen to the results of the study?**

The results of the research will be written up as a report and published and will be shared in relevant humanitarian or public health conferences and with participants themselves if this is something you are interested in. Participants will not be identifiable from these reports.

**Who has reviewed the study?**

This study has been given approval by the University of Aberdeen School of Medicine, Medical Sciences and Nutrition Research Ethics Board.

**What if something goes wrong?**

If you have a concern about any aspect of this study, you should ask to speak to the researchers (contact details below) who will do their best to answer your questions.

**What happens now?**

Thank you for reading this information sheet. Please take time to consider whether or not you think you would like to take part in this study. If you would like further information or ask questions, please do so. If you wish to take part, then we will ask you to sign a consent form.
